# Race Betterment

**Published:** 1914-05

**Authors:** 


### Race Betterment.

That society is fully alive to the present status of our physical and mental welfare is evidenced in the great success of the recent conference held in Battle Creek on Race Betterment.

More than four hundred of our leading men and women of thought met there and discussed the problems that confront the stability of society today. Among the questions considered were tuberculosis and cancer, the increase in crime and insanity, the decreasing birth rate, and alcohol and tobacco as racial poisons.

Race betterment has as its object the development of "mortals free from the mental and physical blemishes of the present day." How this is to be brought about is the great question. In the main, all will agree that preventing the birth of degenerates—feeble-minded, insane, criminals, deaf, blind, epileptic, vagrants—is one means of accomplishing the purpose, but that this method is too drastic and in contradiction to human rights. The more philanthropically inclined rather advocate such measures as will improve the conditions of existing degenerates. This phase of the problem was ably discussed by the president of the conference, Dr. Stephen Smith, who ably presented his conception of the basic principles of race betterment.

He holds that "all efforts to improve the individual, and, through him, the race, must center in the normal development and physiological action of the ultimate elements of the brain, the organ of the mind." Whatever elevates the physiological above the psychological, the body above the mind, is an enemy of the race and no method for its generation. He very properly points out that the cells—the neurons—of which the brain is composed are unified organs, "a self-contained living being," "the sole active principle in every vital function," "the medium of sensation, will and thought, the highest of psychic function." In other words, "as the neurons so is the man." Each cell has its special function and affinity for certain stimulants.

They are influenced by every impression, and are constantly changing. Some cells are never brought into activity and hence do not functionate, while others again are overworked, which accounts for only limited development. The aggregate of these cells constitutes the personality of the individual—"the ego." The degree of their development depends upon conditions personal to the individual. This accounts for the difference in mental development in individuals—the application of the greatest numbers and variety of stimulants to the brain, which we call degeneracy.

Therefore, the doctor asks the question, "How can we make the brain of the defective most useful to its possessor?" or, in other words, "How can we secure to each individual of the race a normal development of his brain cells?" Theoretically, this can be done by a process of mental hygiene, i. e., the control of the function of the brain cells by withholding or increasing the proper stimulant. (This is true barring the limitations placed by heredity.)

The doctor makes a practical demonstration of his position by pointing out the futility and inhuman manner of treating our criminal and degenerate classes. He characterizes such treatment as being barbarous and punitive. It does not individualize or render the person more capable of self-care.

The purpose should be to improve his condition by avoiding all incitements to vice and crime and every possible stimulant to virtue substituted.

As with the criminal so with the insane and feeble-minded. By scientific methods his condition can be made more tolerable if not totally relieved. Thus he states that seventy-five per cent of insanity is curable by proper treatment and that ninety per cent could be made self-supporting. The feeble-minded can be educated to a limited responsibility and even the idiot stimulated to a certain mental activity.

